# TrkB-containing exosomes promote the transfer of glioblastoma aggressiveness to YKL-40-inactivated glioblastoma cells

**DOI:** 10.18632/oncotarget.10387

**Published:** 2016-07-02

**Authors:** Sandra Pinet, Barbara Bessette, Nicolas Vedrenne, Aurélie Lacroix, Laurence Richard, Marie-Odile Jauberteau, Serge Battu, Fabrice Lalloué

**Affiliations:** ^1^ Limoges University, Equipe Accueil 3842, Cellular Homeostasis and Diseases, Faculty of Medicine, 87025 Limoges Cedex, France; ^2^ Limoges University Hospital, Department of Neurology, 87042 Limoges Cedex, France; ^3^ Limoges University Hospital, Department of Immunology, 87042 Limoges Cedex, France; ^4^ Limoges University, Laboratory of Analytical Chemistry and Bromatology, Faculty of Pharmacy, 87025 Limoges, France

**Keywords:** glioblastoma, exosomes, neurotrophin receptors, TrkB, undifferentiated cells

## Abstract

The neurotrophin receptors are known to promote growth and proliferation of glioblastoma cells. Their functions in spreading glioblastoma cell aggressiveness to the microenvironment through exosome release from glioblastoma cells are unknown.

Considering previous reports demonstrating that YKL-40 expression is associated with undifferentiated glioblastoma cancer stem cells, we used YKL-40-silenced cells to modulate the U87-MG differentiated state and their biological aggressiveness. Herein, we demonstrated a relationship between neurotrophin-receptors and YKL-40 expression in undifferentiated cells. Differential functions of cells and derived-exosomes were evidenced according to neurotrophin receptor content and differentiated cell state by comparison with control pLKO cells.

YKL-40 silencing of glioblastoma cells impairs proliferation, neurosphere formation, and their ability to induce endothelial cell (HBMEC) migration. The modulation of differentiated cell state in YKL-40-silenced cells induces a decrease of TrkB, sortilin and p75^NTR^ cellular expressions, associated with a low-aggressiveness phenotype. Interestingly, TrkB expressed in exosomes derived from control cells was undetectable in exosomes from YKL-40 -silenced cells. The transfer of TrkB-containing exosomes in YKL-40-silenced cells contributed to restore cell proliferation and promote endothelial cell activation. Interestingly, in U87 MG xenografted mice, TrkB-depleted exosomes from YKL-40-silenced cells inhibited tumor growth *in vivo*.

These data highlight that TrkB-containing exosomes play a key role in the control of glioblastoma progression and aggressiveness. Furthermore, TrkB expression was detected in exosomes isolated from plasma of glioblastoma patients, suggesting that this receptor may be considered as a new biomarker for glioblastoma diagnosis.

## INTRODUCTION

Glioblastoma (GBM), the most frequent type of malignant tumor in the adult central nervous system, is associated with poor prognosis with a mean survival of 12 months despite advances in surgery, radiotherapy and chemotherapy [1, 2]. Among the hypotheses of therapeutic resistance, YKL-40-expressing cells are identified as mesenchymal cell markers associated to temozolomide resistance [3] and worse prognosis [4]. Additionally, the cellular heterogeneity of tumors containing glioblastoma stem cells (GSC) (also named brain tumor initiating cells) have been reported in GBM [5, 6]. Their ability to remodel actively their microenvironment, contributes to promote tumorigenesis [7]. The exosomes released from tumor cells are described to control the microenvironment. These small bilayer microvesicles (50–150 nm in size) have been extensively studied for their ability to transfer molecular cargo to surrounding cells, influencing the tumor phenotype [8, 9]. Indeed, the transfer of oncogenic receptors such as EGFRvIII in GBM contributes to spread aggressiveness to microenvironment via exosomes [8].

Neurotrophins (NTs), a family of growth factors initially identified in the nervous system [10] are involved in proliferation, differentiation or cell death in several malignant cells. These functions depend on the activation of two types of receptors. Neurotrophin tyrosine kinase receptors, also named tropomyosin kinase (Trk) receptors (TrkA or NTRK1, TrkB or NTRK2, and TrkC or NTRK3), are high affinity receptors for the mature neurotrophins, nerve growth factor (NGF), brain-derived neurotrophic factor (BDNF) and neurotrophin-3 (NT3), respectively. p75^NTR^, a member of TNF receptor superfamily, is the common receptor for mature NTs and their precursors, the pro-NTs [10]. Neurotrophins and their tyrosine kinase receptors are involved in several solid tumor aggressiveness [11]. The overexpression of these receptors was reported in glioblastoma. Indeed, TrkB and TrkC receptors promote the growth of brain tumor-initiating cells [12] and p75^NTR^ was also described in high grade gliomas [13] promoting glioma invasion [14]. Furthermore, TrkB is considered sufficient to transform a neural crest-derived cell line into a malignant phenotype [15]. We have previously described the release of TrkB in exosomes from other malignant cells, the non-small cell lung cancer cells [16]. Interestingly, in this model, the exosome release consisted in the association of TrkB with another oncogenic receptor, EGFR, in presence of sortilin, promoting angiogenesis, proliferation and cell survival [16]. In glioblastoma, the transfer of neurotrophin receptors through exosomes has never been investigated. We hypothesize that the transfer of TrkB through exosomes in GBM may play a key role in spreading aggressiveness and promoting tumorigenesis.

YKL-40 coded by *CHI3L1* gene, a member of the mammalian chitinase-like glycoprotein family is a lectin lacking chitinase activity due to amino acid substitutions in the region corresponding to the chitinase active site. YKL-40 may have a role in cell migration [17] and connective tissue modeling [18] and is involved in the inflammatory response [19, 20]. Additionally, it has been implicated as a serum marker for aggressive disease in colon [21], ovarian [22], breast carcinoma [23] and GBM [24]. Despite the association of increased expression of YKL-40 with many diseases, its biological function is still largely unknown. YKL-40 is involved in growth and survival of glioblastoma cells [25]. YKL-40 is a marker of worse prognosis in high-grade gliomas [26], involved in invasion, angiogenesis [25] and in maintaining the mesenchymal signature of primary glioblastoma [27, 28]. In GBM, elevated serum levels of YKL-40 are positively correlated with cancer invasiveness, radioresistance, recurrence, and reduced patient survival times [4, 24, 29]

We have previously demonstrated that the gene (*CHI3L1*) coding for YKL-40 was overexpressed in undifferentiated cells [30]. In order to define the relationship with neurotrophin-receptor expressions in YKL-40-expressing cells and their derived exosomes, a silencing of YKL-40 in U87-MG cells was achieved to characterize their functional properties.

This study highlights for the first time that YKL-40 silencing induces a decrease of TrkB, sortilin and p75^NTR^ expressions, associated with a low aggressive phenotype. Interestingly, we have evidenced that the release of TrkB in exosomes from control glioma cells, was able to rescue both migration and activation of YKL-40-inactivated cells. By contrast, exosomes from YKL-40-inactivated cells suppressed these functions in control cells.

## RESULTS

### Characterization of YKL-40-inactivated glioblastoma cells: differentiated state and functional properties

YKL-40 coded by *CHI3L1* gene, a member of the chitinase-like glycoprotein family, is involved in growth and survival of glioblastoma cells [25]. We have previously shown that *CHI3L1* is overexpressed in undifferentiated U87-MG cells and in neurosphere-forming glioblastoma stem cells, isolated from GBM patients [30].

**Figure 1:**
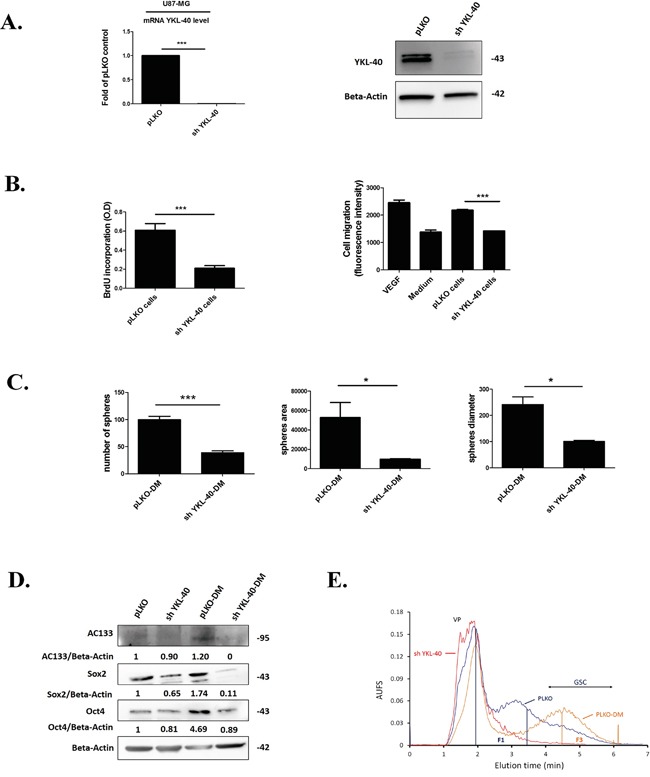
YKL-40 expression is essential to maintain the undifferentiated state cell related to GSC **A.** Quantification of *YKL-40* mRNA by RT-qPCR and protein expression for YKL-40 by western blotting performed in human U87-MG pLKO control cells (pLKO) and sh YKL-40 cells. The fold change of YKL-40 mRNA was normalized by GAPDH and plotted as means ± SD compared to controls. (Student t-test. ***P< 0.001). **B.** BrdU incorporation (left) was performed to analyze proliferation in sh YKL-40 or pLKO cells. Cell (HBMEC) migration assay (right) evaluated by fluorescence intensity was carried out with sh YKL-40 or pLKO cells and compared to VEGF (Vascular Endothelial Growth Factor 10 μg/mL) and culture medium (MEM) as positive and negative controls respectively. **C.** Number, area and diameter of spheres for pLKO-DM and sh YKL-40-DM cells (Student's t-test, *P< .05; ***P< .001). **D.** AC133, Sox2 and Oct4 expression (western blotting) in pLKO, sh YKL-40, pLKO-DM and sh YKL-40-DM. Values indicate the ratio of relative densitometric values of AC133, Sox2 and Oct4 compared to β-actin as loading control, normalized to an arbitrary value of 1 obtained with pLKO cells. **E.** Representative fractograms of pLKO (blue), pLKO-DM (orange) and sh YKL-40 (red) after cell sorting in SdFFF. VP corresponds to Void Peak; Fractions 1 (F1) and 3 (F3) are indicated by vertical lines.

Before studying the neurotrophin receptors expression, we have analysed the functional changes induced by YKL-40 inactivation. To address this, YKL-40 inactivation was performed by shRNA in U87-MG cells. The selected clone was the lower YKL-40-expressing cell line in comparison with pLKO control cells (Figure 1A). This silenced cell line was studied to determine the modulation of undifferentiating cell markers and their functional properties i.e invasion and cell proliferation. According that YKL-40 expression was similar in U87-MG cells and pLKO cells (data not shown), pLKO cells were used as control.

To determine whether the lack of YKL-40 cell expression influences proliferation and migration, invasion assays were performed. Proliferation and capacity to induce HBMEC migration, were significantly decreased in sh YKL-40 cells (*P*=0.0008, *P*=0.0006, respectively) (Figure 1B).

According that stemness properties were maintained in defined medium [31], we have compared sh YKL-40 cells to pLKO cells, cultured in defined medium (DM). The neurosphere-forming cells and the number of undifferentiated cells were evaluated in parallel to YKL-40 mRNA and protein expressions. A lack of YKL-40-expressing cells in DM was observed in sh YKL-40 cells, by contrast to pLKO cells in DM cultures (Supplementary Figure S1). In parallel, the number, surface and diameter of neurospheres were significantly decreased in sh YKL-40-DM cultures compared to pLKO-DM cultures (*P*=0.0010, *P*=0.0384, *P*=0.0108, respectively) (Figure 1C). To search for a relationship of these data with the diminution of undifferentiated cells, we have evaluated the expression levels of the main markers for glioma stem cells (GSC), AC133, Sox2 and Oct4 [31–33]. As expected, GSC markers (AC133 and Oct4) were increased in defined medium (pLKO-DM) compared to pLKO cells cultured in serum-containing medium due to GSCs selection. In contrast, these GSCs markers are reduced when YKL-40-silenced U87-MG cells cultured in defined medium (pLKO-DM) (Figure 1D). Cell sorting by SdFFF confirmed the variation of cancer stem cell content based on the discrimination of differentiated cell sub-population eluted in fraction F1 and undifferentiated cells eluted in fraction F3 [34, 35]. Obviously, F3 is increased in pLKO-DM cell fractogram compared to pLKO cells cultured in serum-containing medium. By contrast, the most undifferentiated sh YKL-40 cells cultured in serum-containing medium cells disappeared in F3 by comparison to PLKO cells and sh YKL-40 cells cultured in DM were undetectable (Figure 1E).

Altogether, these data suggest that YKL-40 inactivation modulates differentiation cell state inducing the disappearance of undifferentiated neurosphere-forming cells.

According that neurotrophin receptors were identified in GSCs and are associated to glioma aggressiveness [12–14] we hypothesized that their expression could be modified in YKL-40-silenced cells. Therefore, we have evaluated the neurotrophin receptors TrkA, TrkB, TrkC, p75^NTR^ and sortilin in YKL-40 inactivated cells, in comparison to control pLKO cells.

### YKL-40 glioma cell-inactivation decreased TrkB, p75^NTR^ and sortilin cell expressions

According that YKL-40 inactivation suppressed the undifferentiated glioma cells and that neurotrophin receptors are associated to the activation of brain tumor-initiating cells [12] we searched for their association with specific changes in the expression level of neurotrophins and their receptors. To address this, we have compared their expressions in YKL-40 silenced U87-MG cells and in pLKO control cells.

**Figure 2:**
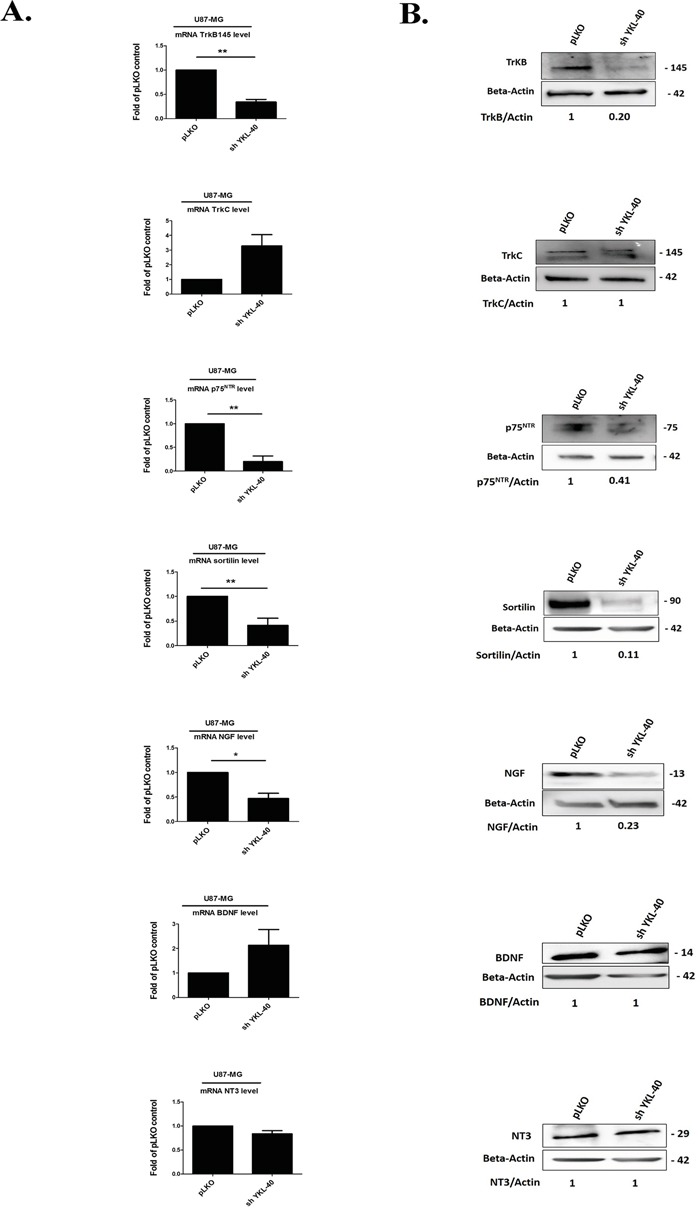
Expressions of neurotrophins and its receptors are modulated by YKL-40 expression level in cells **A.** Analyses of changes in mRNA expression were achieved on different neurotrophin receptors, TrkB, TrkC, p75^NTR^ and co-receptor sortilin, NGF, BDNF and NT-3 by real time PCR in pLKO and sh YKL-40 cells. mRNA values represent fold-change (mean ± SD of three independent experiments) relative to mRNA levels corresponding to pLKO -transfected cells as controls (Student's t-test, *P< .05; **P< .01; *** *P*< .001). **B.** The pattern of protein expression from each gene was studied by western blotting in pLKO and sh YKL-40 cells. Values indicate the ratio of relative densitometric values of TrkB, TrkC, p75^NTR^, sortilin, NGF, BDNF and NT-3 compared to β-actin as loading control, normalized to an arbitrary value of 1 obtained with pLKO control cells. Results are representative of three independent experiments.

Therefore, mRNA and protein expression of both TrkB isoforms (TrkB 145, full-length) and truncated TrkB 95 (Data not shown), p75^NTR^, sortilin and NGF were significantly down-regulated in sh YKL-40 compared to pLKO cells (Figure 2A-2B) while the expression of TrkC, BDNF and NT3 remained unchanged (Figure 2A-2B) and TrkA was undetected whatever the cells (data not shown). Taken together, these data suggested that TrkB, p75^NTR^ and sortilin expressions were related to those of YKL-40.

Accordingly, we next investigate the exosome release from these cells and their contents in neurotrophin receptors.

### Neurotrophin receptors, TrkB, p75^NTR^ and sortilin are decreased in sh YKL-40-derived exosomes

**Figure 3:**
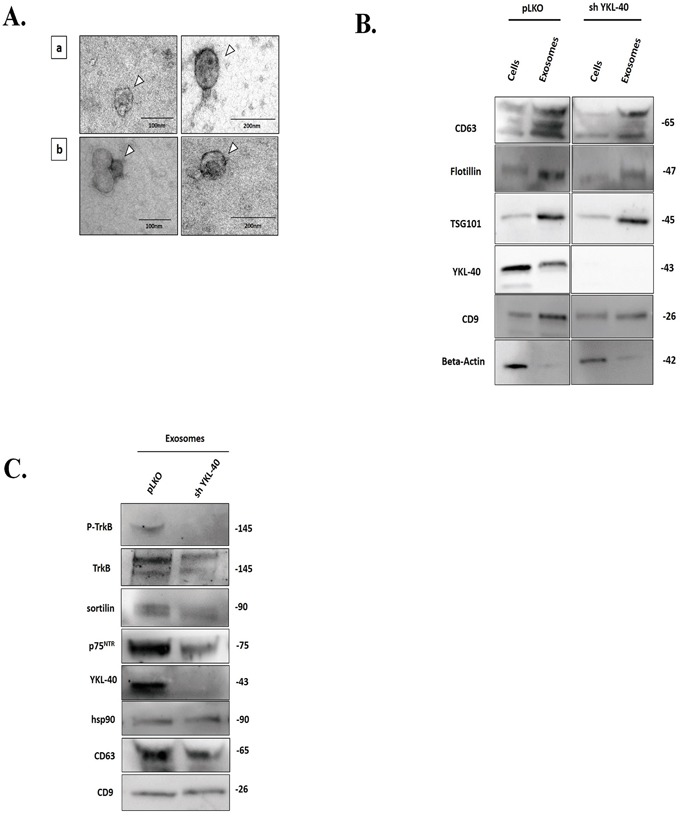
Composition of neurotrophin receptors in exosomes purified from pLKO and sh YKL-40 cells **A.** Electron Microscopy (EM) of nanovesicles, (arrow) obtained after ultracentrifugation (120,000g) of supernatant, validates intact vesicles (40 to 150 nm) derived from pLKO (a) and sh YKL-40 (b) cells, 93000X, scale bar, 100 nm and 200 nm. **B.** Comparison of different marker expression in both cell lines (pLKO and sh YKL-40 cells) and in their derived exosomes. Expression of CD63, Flotillin, TSG101, YKL-40, CD9 and actin was analyzed by western blotting in exosomes and cells for pLKO and sh YKL-40 cell lines. **C.** Comparative expression of P-TrkB, TrkB, sortilin, p75^NTR^, YKL-40 and three exosome markers (HSP90, CD63, and CD9) in pLKO- and sh YKL-40-exosomes by western blotting.

Considering that glioblastoma exosomes are implicated in the activation of tumor growth [36] and that neurotrophin receptors play a key role in glioblastoma aggressiveness, we then analysed their expressions in exosomes isolated from sh YKL-40 cells in comparison to those from pLKO control cells. Isolation of pure and intact exosomes, performed by differential ultracentrifugation protocol [37] were characterized by dynamic light scattering (DLS), transmission electronic microscopy (TEM) and proteomic analysis. DLS analysis showed a similar size between exosomes released from pLKO cells (pLKO exosomes) (90.0 ± 9.4 nm) and those from sh YKL-40 cells (sh YKL-40 exosomes) (80.0 ± 19.6 nm) (Supplementary Figure S2). TEM data confirmed that two cell types released a homogenous mixture of rounded vesicles (Figure 3A).

Main exosome markers were found in nanovesicles derived from both cell subtypes (Figure 3B). Our results demonstrated that exosome structure and specific markers are conserved in both cell types.

The specific expression of YKL-40, TrkA, TrkB, TrkC, p75^NTR^ and sortilin was studied by western blotting in exosomes derived from pLKO cells and from sh YKL-40 cells cultured in exosome-free medium. As expected, YKL-40 expression was undetectable in sh YKL-40 exosomes. TrkB, p75^NTR^ and sortilin receptors were down-regulated in sh YKL-40 exosomes compared to pLKO exosomes (Figure 3C). Strikingly, phosphorylated TrkB was detected only in exosomes from pLKO cells (Figure 3C). Similarly to cell expression, TrkA was undetected and TrkC was unchanged in both cell line-derived exosomes (Supplementary Figure S3). As EGFRvIII was detected in exosome released from glioblastoma cells [8], we have studied in parallel its expression. Interestingly, we have detected that EGFRvIII was also decreased in exosomes released from sh YKL-40 cells, compared to pLKO cells (Supplementary Figure S3). To specifically determine the differential distribution of neurotrophin receptors and YKL-40 within exosome subpopulations, protein analyses were achieved on fractions after sucrose gradient separation [37, 38] (0.2-2.5M/density: 1.06-1.21 g/mL) of total exosomes validated by CD9 and CD63 expression (Supplementary Figure S4). The pattern of protein expression in fractions was distinct in the two cell lines. Concerning pLKO exosomes, YKL-40, TrkB, sortilin and p75^NTR^ was mainly expressed in the high-density fractions of 1.16–1.21 g/mL. By contrast, in exosomes derived from shYKL-40 cells, YKL-40, TrkB, sortilin and p75^NTR^ expression were absent (Supplementary Figure S4).

Taken together, these data assessed that inactivation of YKL-40 in glioma cell consistently reduced TrkB, p75^NTR^ and sortilin in exosomes. Therefore, we have looked for their functional effects in proliferation and invasion properties.

### Functional properties of exosomes were depending on the transfer of TrkB

Since exosome-derived from cancer cells can promote angiogenesis and tumor growth [16, 39], the effect of pLKO and sh YKL-40 exosomes on glioma cell proliferation and HBMEC migration were analyzed. Moreover, the expression levels of TrkB and phospho-TrkB following exosome internalization were evaluated in both cell lines. For these experiments, cross treatments were performed. Hence, pLKO cells were treated by sh YKL-40 exosomes (30μg/mL) for 24 hours, whereas sh YKL-40 cells were treated by pLKO exosomes (30μg/mL) in same conditions. Each condition was compared to untreated cells and pLKO cells treated by pLKO exosomes as controls.

**Figure 4:**
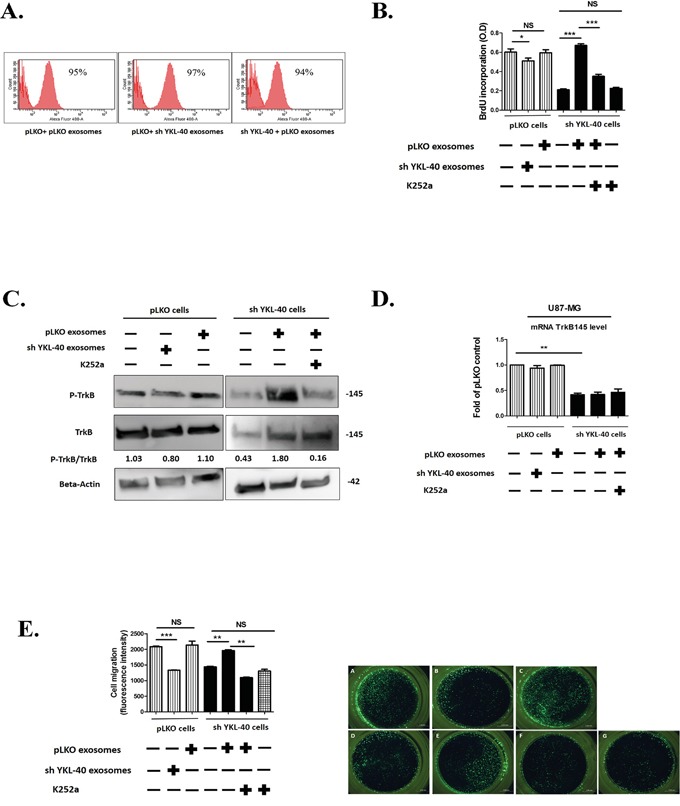
Functional properties of pLKO and sh YKL-40-exosomes **A.** Internalisation of pLKO or sh YKL-40 exosomes (30μg/mL) in recipient cells. The exosome uptake was quantified by using labelled exosomes with PKH67 at the same concentration for 24h and was analysed by flow cytometry. **B.** A representative experiment of proliferation analyzed by BrdU incorporation in sh YKL-40 or control pLKO cells after a 24-hour co-culture with 30μg of pLKO- or sh YKL-40-derived exosomes with or without 100 nM of K252a. Histograms were representative of three independent experiments (Student's t-test, *P< 0.05; *** P<0.001) **C.** Expression of phospho-TrkB and TrkB proteins in pLKO or sh YKL-40 cells after a 24-hour co-culture with 30μg of pLKO or sh YKL-40 exosomes with or without treatment of 100 nM of K252a. **D.** Quantification of TrkB mRNA (qRT-PCR) in pLKO or sh YKL-40 cells in presence of exosomes in same experimental conditions as in C. Data were normalized to GAPDH and plotted as means ± SD compared to controls (Student's t-test, **P<.01). **E.** Right part: Invasion assay were used to detect HBMEC migration. HBMEC cells (15,000/well) were added to the upper chamber. In the lower chamber, we placed 75,000 cells/well of either pLKO cells alone (A), or pretreated for 24 h with 30μg of sh YKL-40-exosomes (B) or pretreated for 24 h with 30μg of pLKO-exosomes (C) or sh YKL-40 cells alone (D), or pretreated for 24 h with 30μg of pLKO-exosomes (E, F) with 100 nM of K252a. (F, G) After 18 hours, the number of HBMEC cells that migrated to the lower chamber through the 8μm pore-sized membrane was analyzed by taking photos and counting the number of cells per visual field. Left part: Histograms representing five independent experiments. (Student's t-test *P< .05; **P<.01; ***P<.001).

Internalization of exosomes in recipient cells was visualized by using exosomes labelled with green fluorescent dye PKH67 [40] and quantified by flow cytometry. In each condition, exosomes (30μg/mL) were uptaken as soon as 30 minutes after their addition to reach a maximum for both at 24 hours (Figure 4A).

To investigate the effects of exosomes on cell proliferation, BrdU was incorporated to cell culture (Figure 4B). As shown previously in Figure 1B, without exosome treatment, cell proliferation significantly decreased in sh YKL-40 cells compared to pLKO cells (*P*=0.0008). In contrast, proliferation was restored in sh YKL-40 cells treated by pLKO exosomes (*P*= 0.0005). Conversely, the proliferation of pLKO cells treated by sh YKL-40 exosomes significantly decreased (*P*=0.0458), demonstrating that the induction of proliferation may depend on exosome origin. Interestingly, proliferation of sh YKL-40 cells treated with pLKO exosomes was significantly decreased by addition of K252a, a tyrosine kinase inhibitor for Trks [41] (Figure 4B, *P*=0.0006). This result suggests that cell proliferation induced by the exosome transfer depends on tyrosine kinase neurotrophic receptors.

Given the role of TrkB in the growth of GSCs and glioblastoma cells [12], we next studied TrkB expression and phosphorylation in pLKO and sh YKL-40 cells treated or not by exosomes. TrkB protein expression was increased in sh YKL-40 cells treated by pLKO exosomes (Figure 4C) without increase of TrkB transcripts (Figure 4D) assessing that TrkB expression resulted in exosome protein transfer (Supplementary Figure S5). In contrast, TrkB expression was unchanged when pLKO cells were treated with sh YKL-40 exosomes (Figure 4C). Remarkably, TrkB was phosphorylated in sh YKL-40 cells exposed to exosomes from pLKO cells. This activation was suppressed by k252a inhibitor (Figure 4C), supporting that exosomes induced TrkB activation in recipient cells. In contrast, TrkB activation in pLKO cells was undetectable when exosomes were isolated from sh YKL-40 cells (Figure 4C).

Without exosome treatment, sh YKL-40 cells significantly decreased HBMEC migration compared to pLKO cells (*P*= 0.0006). Then, we searched for the exosome capacity to rescue the lack of migratory effect of sh YKL-40 on HBMEC migration in a Boyden chamber (Figure 4E). Interestingly, sh YKL-40 cells pretreated with pLKO exosomes before insertion in lower chamber, restored the migration of HBMEC (*P*= 0.0022). In addition, the role of TrkB activation in inducing HBMEC migration is supported by the inhibition of migration by the concomitant incubation with K252a Trk inhibitor (*P*= 0.0023). As observed above for cell proliferation, HBMEC migration was significantly decreased in pLKO cells pretreated with sh YKL-40 exosomes (*P*= 0.0004) (Figure 4E). These results suggest that pLKO exosomes restored the functional properties (proliferation and endothelial cell migration) of sh YKL-40 cells, through the transfer and activation of TrkB, and that inactivated cells for YKL-40 released exosomes with suppressive functions.

Considering the previous *in vitro* results, we have studied the effect of the two types of exosomes on tumor growth in mice.

### Tumor growth was diminished by exosomes from YKL-40-inactivated cells

Experimental tumors were developed by subcutaneous injections of pLKO or sh YKL-40 cells into one flank of SCID mice. For each group, 15 mice were used. After one week, pLKO or sh YKL-40 xenografts were treated twice a week, during three weeks, by pLKO exosomes, sh YKL-40 exosomes or PBS as control. To evaluate the effects of exosomes in recipient tumors and avoid spreading and accumulation in other tissues [42], exosomes (30μg/mL) were injected in hydrogel suspension closely to the tumor site, to allow local and progressive exosome release.

**Figure 5:**
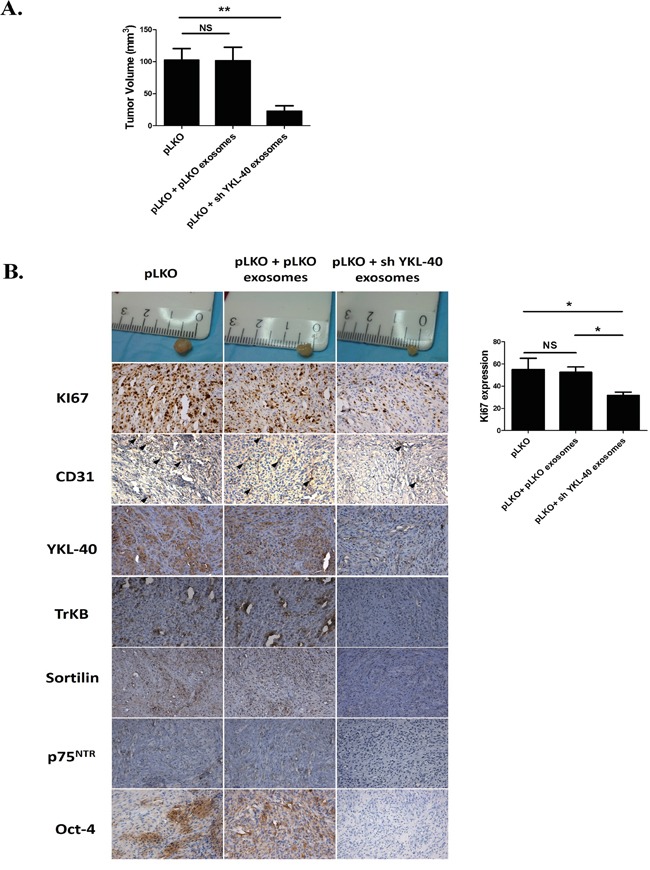
SCID mouse heterotopic xenograft model. **A.** pLKO cells were subcutaneously injected into SCID mice (n=15 mice per group) Tumors were developed at week 1. One week after pLKO cell xenografts, animals were treated by local injection with PBS/hydrogel (control) or 30μg of pLKO or sh YKL-40 exosomes/hydrogel twice per week during three weeks. Tumor volume and tumor weights were determined at the end of the treatment. Data are presented as the mean ± SD, **P<.01 compared to PBS controls by ANOVA. **B.** Proliferation visualized by Ki67 staining decreased in pLKO-derived tumor treated by sh YKL-40 exosomes. Immunohistochemical stainings performed in xenograft tumors showed a similar decrease in TrkB, Sortilin, p75^NTR^, Oct-4 and endothelial marker CD31 expression. Scale Bar, 50μm.

When sh YKL-40 cells were injected, we failed to obtain any tumor, whatever the amount of graft cells or exosome treatment (data not shown). In contrast, pLKO cells induced tumors with a mean volume of 100 mm^3^ (Figure 5A). Tumor volumes were unchanged after treatment by pLKO exosomes (Figure 5A). Strikingly, pLKO tumors treated with sh YKL-40 exosomes were significantly smaller (Figure 5A; *P*=0.0070). In addition, cell proliferation, evaluated by Ki67 staining, was diminished in pLKO tumors treated by sh YKL-40 exosomes, compared to untreated pLKO tumors (Figure 5B). These data are consistent with those obtained for *in vitro* proliferation experiments (Figure 4B).

In addition, several markers related to tumor angiogenesis (CD31) [43] or to undifferentiated state cell (YKL-40 and Oct-4) were analysed in xenografts (Figure 5B). Expression of main markers was unchanged after treatment of tumors by pLKO exosomes. However, CD31 expression decreased in pLKO tumors treated by sh YKL-40 exosomes, suggesting that tumor angiogenesis was inhibited by exosome treatment. Both YKL-40 and Oct-4 expressions decreased in pLKO tumors treated by sh YKL-40 exosomes compared to untreated tumors. To determine whether expression of neurotrophin receptors in tumors could depend on exosomes content, TrkB, p75^NTR^ and sortilin expressions were evaluated. While *in vitro* TrkB remains detectable (Figure 4C), its expression was clearly reduced in pLKO tumors treated by sh YKL-40 exosomes. A similar decrease was observed for p75^NTR^ and sortilin. Altogether, these data demonstrated sh YKL-40 cell exosomes reduced tumor growth and undifferentiated markers (YKL-40 and Oct-4) as well as TrkB, p75^NTR^ and sortilin expressions.

According that TrkB expression was detected in exosomes from glioblastoma cells in cultures; we have extended this study to exosomes from glioblastoma patients.

### TrkB is detected in plasma exosomes from GBM patients

**Figure 6:**
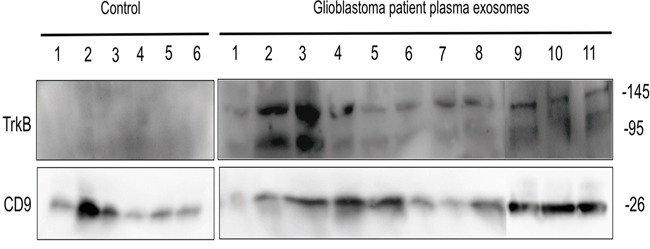
TrkB expression in exososmes from GBM patients Exosomes isolated from GBM patients plasma (n=11) and age-matched control subjects (n=6) were analyzed for TrkB by western blotting. CD9 exosome marker was used as loading control.

Given that exosomes are accessible from body fluids, it could represent a promising source of biomarkers for cancer diagnosis [44]. We have determined whether TrkB detection in exosomes isolated from plasma GBM patients could constituted a new biomarker of GBM. Exosomes were purified by ultracentrifugation from plasma of GBM patients (n = 11) and healthy donors (n=6) before analyzing TrkB and CD9 expression. Exosomes were characterized by CD9 expression, a tetraspanin specifically enriched in their membrane. Interestingly, we found that in all cases (n=11), both TrkB isoforms were detected in exosomes from GBM patients and totally absent in the age-matched controls (n=6) (Figure 6). It is noteworthy that TrkB expression levels varied in patients' exosomes, some of them exhibiting a high overexpression (Figure 6).

## DISCUSSION

This study supports a new function of glioblastoma-derived exosomes in the transfer of neurotrophin receptors to promote glioma cell aggressiveness. Several studies have already reported the oncogenic functions on neurotrophin receptors, overexpressed in several cancers such as glioblastoma [12, 14, 45]. Neurotrophin signalling also contributes to tumor cells and GSCs survival and growth promoting aggressiveness and therapeutic resistance [12]. The importance of exosomes in the spreading of biological aggressiveness was reported in prostate cancer cells in which exosomes enhance invasiveness, stemness and induce microenvironment changes [46, 47]. Since our previous findings have demonstrated that TrkB is expressed in exosomes from lung cancer cells [16], we hypothesized that the transfer of TrkB through exosomes in GBM may play a key role to spread aggressiveness in the microenvironment and promote tumorigenesis. Additionally, the content of exosome released by stem cells depends on cell differentiation [48].

In order to study the loss of tumor aggressiveness, *YKL-40*, a gene coding for a cancer stem cell marker [30, 49] was inactivated in glioblastoma cells. Its silencing induced both a decrease of cell proliferation as previously shown [30] and HBMEC migration confirming the role of YKL-40 in the control of vascular endothelial cells and tumor angiogenesis [25, 50, 51]. Moreover, YKL-40 silencing led to a significant decrease of neurosphere-forming cells as well as a loss of the main markers of GSCs. These data correlated with the cell-sorting elution profile of sh YKL-40 cells, confirming the loss of undifferentiated cells. These results supported stemness properties of YKL-40 [49], considered as a mesenchymal marker expressed in high grade glioma [46]. Altogether, these data suggested that the decrease of these functional properties is closely linked to the differentiation state of YKL-40-silenced cells. The impairment of proliferation, induction of HBMEC migration and neurosphere-forming cells in sh YKL-40 cells, suggest that these cells acquired a low aggressive phenotype.

This loss of aggressiveness in YKL-40-silenced cells also influences neurotrophins and neurotrophin-receptor expression. Indeed, YKL-40 silencing significantly decreased TrkB, p75^NTR^ and sortilin expression. These data demonstrate that neurotrophin receptors expression also depend on YKL-40-expressing cells and related to their differentiated state. Their reduced expressions could also prevent their major role in GSC proliferation and glioblastoma growth [12, 47]. Such results were not previously described. We hypothesize that YKL-40 may play a putative role on the regulation of neurotrophin receptors expression and GBM cells activation. Indeed, the relationship between YKL-40 and neurotrophins has been already reported in peripheral nervous system. In this tissue, NGF (Nerve growth factor) stimulation induces increased expression of YKL-40 suggesting that NGF has potential effects on matrix turnover activity [52]. This relationship could be related to the decreases neurotrophin receptor expression and activation induced by YKL-40 silencing in U87-MG cells. Furthermore, the analysis of glioblastoma secretome revealed the presence of YKL-40 secreted by glioma stem cells, able to induce malign transformation of normal neural precursor cells [53]. These previous data suggest that YKL-40 could regulate neurotrophin receptors expression and GBM cells activation.

The protein content of exosomes was modified by YKL-40 silencing and could alter the intercellular transfer of oncogenic receptors between cells, as previously reported for EGFRvIII in glioblastoma [8, 36]. Similarly, the expression of neurotrophin receptors, TrkB, p75^NTR^ and sortilin, were decreased in sh YKL-40-derived exosomes. Interestingly, exosomes from pLKO cells contained phosphorylated TrkB and not sh YKL-40 exosomes. A similar transfer of phosphorylated oncogenic receptor through exosomes was previously described for EGFR in glioma cells [54].

To determine whether TrkB receptor expression in exosomes could modify the activation of surrounding cells and communication with microenvironment, recipient cells were treated with distinct exosomes. Whatever the recipient cells, the exosomes were internalized. Treatment of sh YKL-40 cells with pLKO exosomes significantly restored their proliferation and their ability to enhance HBMEC migration compared to sh YKL-40 cells alone. By contrast, both mechanisms were affected in PLKO cells treated by sh YKL-40 exosomes, depleted in TrkB. Thus, we hypothesized that TrkB receptors contained in pLKO exosomes could re-establish the biological functions of sh YKL-40 cells. TrkB contained in pLKO exosomes could support these functions. Indeed, inhibition of functional rescue by K252a, a pan-Trk inhibitor [41], inhibiting TrkB activation, suggested that TrkB is implicated in these mechanisms, since TrkA was absent and TrkC was unchanged whatever cell types. TrkB mRNA expression level was unchanged in sh YKL-40 cells suggesting that the increase of TrkB receptor into sh YKL-40 cells is mainly due to its protein transfer through exosomes as already demonstrated with c-kit [55]. Furthermore, the transfer of TrkB may affect recipient cell signalling, since TrkB phosphorylation was upregulated in sh YKL-40 cells treated with pLKO exosomes. A similar mechanism has been reported with c-kit-containing exosomes that activate proliferation and PI3K dependent-signalling pathway [55]. Moreover, TrkB phosphorylation was slightly reduced by treatment of pLKO cells with sh YKL-40 exosomes. These results point out a new mechanism, depending on the transfer of TrkB-containing exosomes, able to spread aggressiveness and invasiveness to surrounding cells.

Xenografts in mice showed that YKL-40-inactivated cells failed to induce tumors whatever the amount of grafted cells. These data correlated with previous reports showing that YKL-40-neutralizing-antibody treatment blocks xenograft tumor growth [56]. Herein, we validated that U87-MG cells inactivated for YKL-40 lose their tumorigenic properties due to the lack of aggressive GSCs [57]. Thus, the cross transfer of tumorigenic activity by pLKO exosomes could not be determined in this condition.

However, tumors induced by pLKO cells treated by sh YKL-40 exosomes significantly reduced their volumes, proliferation rates and vascularization (decrease of CD31 expression) suggesting that exosome contents may inhibit tumorigenic mechanisms by modulating angiogenesis. These data correlated with previous study demonstrating that anti-YKL-40 antibodies inhibited angiogenesis in glioblastoma [58] supporting the functions YKL-40 in glioblastoma vascularization [51]. The inhibition of tumor angiogenesis through exosomes from mesenchymal stem cells, was also associated to a down-regulation of VEGF expression [59]. By comparison to sh YKL-40, pLKO tumor growth was unchanged after treatment with pLKO exosomes, suggesting that the additional effect on tumorigenic mechanisms is specific to YKL-40-inactivated cells. The lack of neurotrophin receptors in sh YKL-40 exosomes as shown *in vitro*, was not sufficient to explain the reduction of YKL-40, TrkB, p75^NTR^ and sortilin expression in treated pLKO xenografts. Indeed, the repeated addition of sh YKL-40 exosomes derived from their inactivated cells, seemed to downregulate cell proliferation and modulate differentiated cell state in regards to Oct4, a specific GSC marker and YKL-40 expression depletion. Among the different microRNA identified in GBM, one of them, miR-128a, is associated to the down-regulation of YKL-40 expression in U 87-MG cells [28]. Such mechanisms depending on miRNA could be hypothesized. Indeed, the tumor growth inhibition could also depend on variation contents of specific miRNA transferred by sh YKL-40 exosomes.

In conclusion, we revealed the essential role of exosomes in communication between cancer cells and surrounding stroma and demonstrated that TrkB expression in exosomes is required for inducing aggressiveness phenotype. A similar mechanism was already described with the transfer of activated oncogenic tyrosine kinases receptor, EGFRvIII [8]. In this study, we highlighted that according to the differentiation state and YKL-40 expression, cancer cell-derived exosomes display different neurotrophin expression in glioblastoma. TrkB transfer depends on exosomes, which could contribute to spread the aggressive phenotype to surrounding cells into the microenvironment. Such a mechanism might play a key role in a several types of human tumors. Since our results indicate that TrkB is detected in exosomes from plasma patients, its potential role as a biomarker should be assessed on a large panel of glioblastoma patients and its expression compared according to the glioma grade.

## MATERIALS AND METHODS

### Cell lines

Human GBM cell lines U87-MG were purchased from American Type Culture Collection (ATCC). Three different lentiviral plasmids (pLKO.1, Sigma-Aldrich) containing shRNA sequences (Sigma-Aldrich) to *YKL-40 gene* were used to generate a stable YKL-40 silenced U87-MG cell line using the packaging cell line HEK-293T [16]. HEK-293T cells were transfected by JetPEI and pLKO-YKL-40 or empty pLKO plasmids (Invitrogen, Life Technologies). Lentivirus particles were added to U87-MG, and after 48 h infected cells were selected with puromycin (1μg/mL) and YKL-40 silencing was controlled by qPCR and western blotting analysis for each clones (data not shown).

Two derived cell lines from U87-MG cells were used in this study: human empty vector pLKO control cells and sh YKL-40 cells. Human Brain Microvascular Endothelial Cells (HBMECs, Lonza) were cultured as described previously [16].

### Cell culture

pLKO and sh YKL-40 cell lines were grown in complete medium containing Minimum Essential Medium (MEM) (Lonza) supplemented with 10% FBS (Life Technologies), 2% sodium bicarbonate, 1% sodium pyruvate, 1% non-essential amino acids solution and 1% penicillin/streptomycin at 37 °C in a humidified atmosphere of 5% CO_2_ and 95% air.

To obtain neurospheres, sh YKL-40 cells and pLKO cells were grown in defined medium (serum-free neural stem cell medium) corresponding to Neurobasal/Glutamax (Invitrogen) supplemented with 1% N2 and 2% B27 (Invitrogen) and 20 ng/mL epidermal growth factor and fibroblast growth factor–2 (PeproTech) [34, 60]. After a 4-day culture, both cell lines formed primary neurospheres. Primary neurospheres were dissociated and sh YKL-40 cells (sh YKL-40-DM) or pLKO cells (pLKO-DM) were seeded to form secondary neurospheres.

Treatments with a Pan –Trk inhibitor k252a (100 nM) or dimethyl sulfoxide (DMSO) for 24 h were performed on 1.5 × 10^5^ cells cultured in 6-well plates.

### Quantitative reverse transcription-PCR (qRT-PCR)

Qiagen RNeasy kit (Qiagen) was used to isolate total RNA from cells at day 15 after lentiviral infection as previously described [16]. Briefly, 100 ng cDNA was used for each PCR reaction, performed with TaqMan on ABI Step One Plus real-time thermal cycler (Applied Biosystems). PCR primers for both full-length and truncated form of TrkB, sortilin, TrkC, p75^NTR^, NGF, BDNF, NT3, YKL-40 and GAPDH mRNA were designed and used for PCR amplification with Taq DNA polymerase (Roche Diagnostics) (Supplementary Table S1).

### Western blotting analysis

Cells and exosomes were lysed in lysis buffer [50 mM Tris-Cl (pH 8.0), 150 mM NaCl, 1% NP-40, 0.5% sodium deoxycholate, 0.1% SDS, 100μg/ml phenylmethylsulphonyl fluoride, 0.5 μg/ml leupeptin, and 1 μg/ml aprotinin]. Protein concentrations were determined using a Micro BCA™ protein assay kit (Pierce Biotechnology). Equal amounts of protein were separated by sodium dodecyl sulfate polyacrylamide gel electrophoresis and transferred to PVDF membranes. Membranes were blocked with 5% skim milk in TBS containing 0.1% Tween-20 for 2 h at room temperature and incubated with the appropriate primary and secondary antibodies. Antibodies against CD63 (HPA010088, 1/1000) and β-actin (A 5316, 1/10000) were purchased from Sigma-Aldrich. Antibodies against YKL40 (ab77528, 1/1000), sortilin (ab16640, 1/1000), CD9 (ab92726, 1/1000) and HSP90 (ab13492, 1/1000), TSG101 (ab30871, 1/1000) were obtained from Abcam. Antibodies against TrkA (AF175, 1/200) TrkB (MAB397, 1/200), TrkC(AF1404, 1/200) and P75^NTR^ (AF1157, 1/1000) were purchased from R&D System. Antibodies against NT3 (5237SC, 1/1000), Sox2 (3579S, 1/1000), Oct4 (2750S, 1/1000) and flotillin (18634S, 1/1000) were purchased from Cell Signaling Technology. Antibodies against NGF (sc-548, 1/200) and BDNF (sc-20981, 1/200) were purchased from Santa Cruz Biotechnology.

### Cell sorting method

The cell sorting was performed by an antibody-free elution method based on Sedimentation field-flow fractionation (SdFFF) method, able to isolate undifferentiated glioma stem cells as previously described [34]. SdFFF is a gentle and non-invasive cell sorting method by limiting cell-solid phase interactions by the use of 1) an empty ribbon-like channel without a stationary phase; and 2) the “hyperlayer” elution mode, a size/density driven separation mechanism. The, large and less dense cells are focused in the faster streamlines and are eluted in first fraction (F1) while the small and dense cells are eluted in last fraction (F3) [35, 61]. Cell sorting was realized with pLKO or sh YKL-40 cells cultured in FCS-containing medium as well as in defined-medium.

### Exosomes purification and characterization

Exosomes were isolated from pLKO and sh YKL-40 cells cultured at 37°C in a humidified 5% CO2 atmosphere in complete medium (MEM with 5% exosome-free FCS, previously centrifuged for 16 h at 120,000 x g to remove bovine exosomes). Cell supernatants were harvested after 48 h in culture to purify exosomes by differential centrifugation [37]. Briefly, supernatants were firstly centrifuged at 16,500 x g for 30 min at 4°C. Next, exosomes were pelleted by ultracentrifugation at 100,000 x g for 80 min at 4°C, washed in PBS and re-centrifuged at 100,000 x g for 2 h at 4°C. For each exosome preparation, the concentration of total proteins was quantified by BioRad protein assay kit (Bio-Rad, Hercules, CA).

Exosomes control by electron microscopy: vesicle suspensions for electron microscopy were further diluted 1:10 with PBS and 5μl suspension was applied to glow-discharged formvar-carbon films on copper 200 mesh Ni (Agar scientific). The adsorbed exosomes were negatively stained with 1% aqueous uranyl acetate. The samples were examined with a Jeol 1011 electron microscope (JEOL) equipped with an AMT XR60B digital camera (Advanced Microscopy Techniques).

Exosomes conditioning cell cultures: To study effects of exosomes released from the two cell types on recipient cells, 30μg/mL of exosomes derived from pLKO cells were added to shYKL-40 cells. Inversely, 30μg/mL of exosomes derived from shYKL-40 cells were added to pLKO cells. After 24 hours, proliferation, migration assays and western blotting were performed.

Internalization of exosomes in recipient cells was visualized by using exosomes labelled with green fluorescent dye PKH67 [40] and quantified by flow cytometry.

### Separation of vesicles on sucrose gradient

Exosomes purified from pLKO and sh YKL-40 cells were resuspended in 400 μL PBS-2.5M sucrose, loaded in a SW41 tube and overlaid with 15 successive 600 μl layers of PBS containing decreasing concentrations of sucrose (from 2.5 to 0.2 M). Tubes were centrifuged for 16 hours at 4°C at 200,000 g. 1 mL fractions were collected; sucrose density was measured on an aliquot of each on a refractometer, and fractions were diluted in 2 ml PBS, ultracentrifuged at 100,000 g for 1 hour and resuspended in lysis buffer before analysis by Western blotting [38].

### Proliferation assays

BrdU cell proliferation assays (Calbiochem) were carried out according to the manufacturer's protocol. After a 24-h cell exposure to exosomes, BrdU was added for 4 h to the culture medium before measurement of absorbance at dual wavelengths of 450 and 590 nm.

### Invasion assays

Invasion assays were performed in BD Biocoat Matrigel 24-well plates (BD Biosciences). Briefly, 25×10^3^ HBMECs were seeded in a transwell insert. Assays were performed by inserting pLKO or sh YKL-40 cells in the lower chamber or exosomes derived from these cells. Then, pLKO cells previously treated with 30μg/mL sh YKL-40 exosomes for 24h, or sh YKL-40 cells treated with 30μg/mL pLKO exosomes for 24h, were added to the lower chamber and incubated with HBMEC cells during 20 h at 37 °C / 5% CO_2_. Then, HBMEC in the upper chamber were stained with 50μM calcein AM (BD Biosciences) for 30 min. Non-invading cells in the upper part of the insert were carefully removed. Cells were seeded in triplicate for each cell line. Five independent assays were performed. Invading cells were visualized using a fluorescence microscope MZFL3 (Leica). Fluorescence intensity related to cell density by well was quantified by ImageJ. Histograms are presented as means ± SD.

### Tumor xenografts in mice

Female NOD/SCID mice (Janvier, Saint Berthevin, France) were injected subcutaneously with 1×10^6^ pLKO or sh YKL-40 cells in 50μL of PBS. Three groups of 15 mice per group were treated one week after cell injection with either PBS/hydrogel (controls), 30μg/mL pLKO exosomes/hydrogel or 30μg/mL sh YKL-40 exosomes/hydrogel, bi-weekly for three weeks. Mice were evaluated bi-weekly to measure tumor volumes (V= 0.5 (length × width^2^) before sacrificing the animals (n=37). The experimental procedures were in accordance with the guidelines of Institutional Animal Care and the French National Ethics Committee. All comparisons between groups were performed by ANOVA using Statview 5.0 (Abacus Concepts). Differences were considered significant at *P*<0.05.

### Immunohistochemistry

Animals were sacrificed after four weeks and tumors were removed. Hematoxylin–eosin (H&E) staining and immunohistochemistry were performed on 4μm serial coronal sections from paraffin-embedded tumors. Tissue sections were prepared as previously described [62]. Tissue sections were incubated with antibodies against human Ki67 (1/50, Dako), CD31(1/30, Histonova), YKL-40 (1/1000, Abcam), TrkB (1/200, R&D), p75^NTR^ (1/300, Santa Cruz), sortilin (1/1000, BD bioscience) and Oct4 (1/200) according to the manufacturer's instructions (Boster Bioengineering Company Limited). Anti-rabbit (1/1000) and anti-mouse (1/1000) immunoglobulins HRP EnVision™+ system (Dakocytomation, Glostrup) and DAB (DakoCytomation) were used for the staining revelation.

### Collection of plasma from glioblastoma patients

Plasma samples from 11 confirmed glioblastoma patients before brain tumor surgery and 6 age-matched controls were collected using the same protocol [36] after informed consent and approval by the Ethics Committee of the Limoges University Hospital (Protocol 141-2014-08). These samples were kept at −80°C until use.

### Statistical analysis

Treatments, proliferation assay, relative fluorescence intensities, RT-qPCR and western blotting experiments were compared to controls using Statview 5.0 software. Data are presented as means +/− SD for at least three independent experiments. Comparisons between groups were analyzed by ANOVA or Student's t-test. *P*<0.05 was considered significant (*P<.05; **P<.01; ***P<.001).

## SUPPLEMENTARY MATERIALS FIGURES AND TABLES




