# Supplementary material for: TrkB-containing exosomes promote the transfer of glioblastoma aggressiveness to YKL-40-inactivated glioblastoma cells
Source: Oncotarget. 2016 Jul 2;7(31):50349–64. doi: 10.18632/oncotarget.10387 (PMC5226587; doi:10.18632/oncotarget.10387)
Supplement: Supplementary file 1 [file oncotarget-07-50349-s001.pdf]

TrkB-containing exosomes promote the transfer of glioblastoma aggressiveness to YKL-40-inactivated glioblastoma cells

SUPPLEMENTARY FIGURES AND TABLE

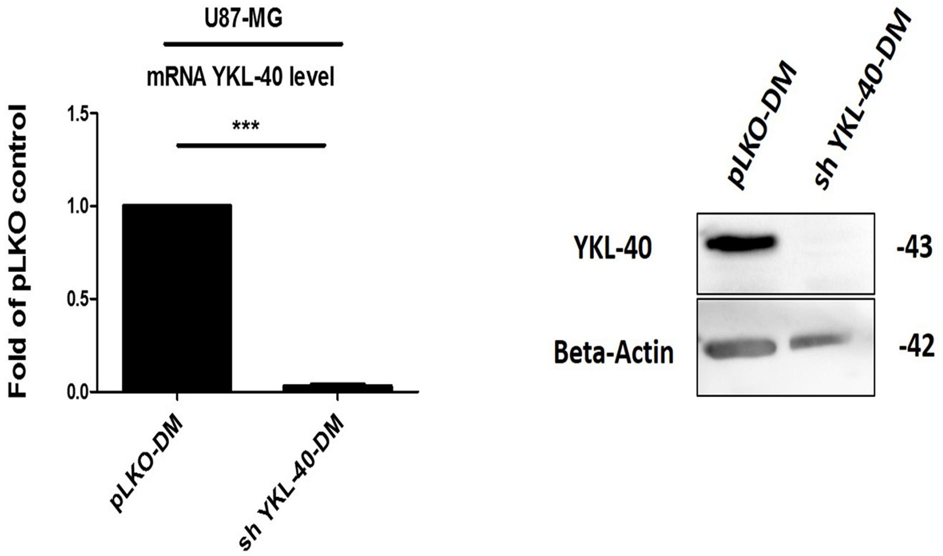

Supplementary Figure S1: Expression of YKL-40 in pLKO and sh YKL-40 cultured in defined medium. Quantification of *YKL-40* mRNA (qRT-PCR) and protein expression (Western blot) in pLKO, sh YKL-40 cultured in defined medium (pLKO-DM and sh YKL-40-DM). Data were normalized to GAPDH and plotted as means ± SD compared to controls (Student's t-test, ****P*<.001). Western blotting was normalized to β-actin.

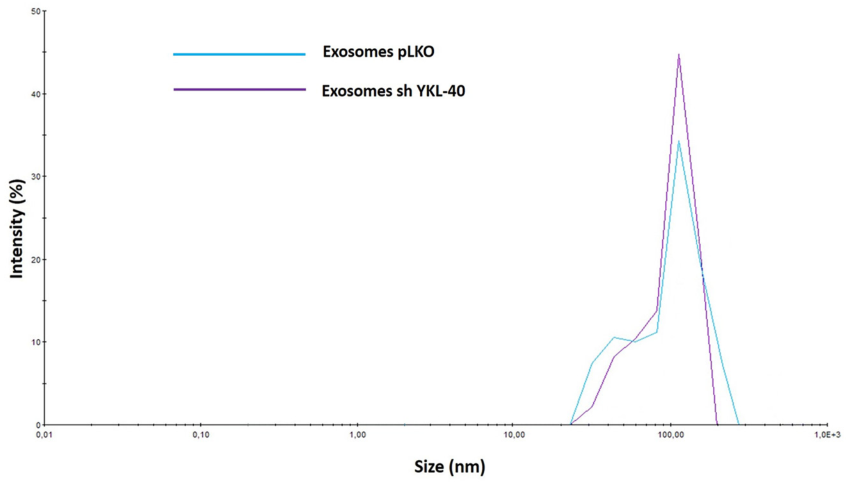

Supplementary Figure S2: Particle size distributions of exosomes isolated from pLKO and sh YKL-40. Particle size distributions of exosomes isolated from pLKO (blue) and sh YKL-40 (purple) as measured by Dynamic Light Scattering (DLS) system coupled with dynamics v7 (Wyatt technology, Santa Barbara,CA). Intensity is related to counted particle number.

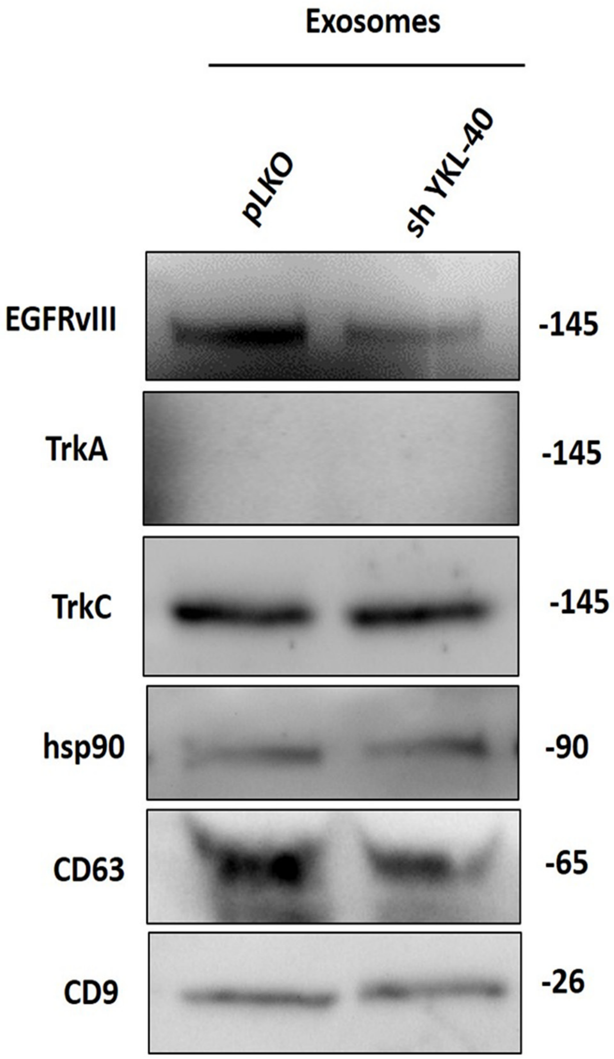

Supplementary Figure S3: Expression of EGFRvIII, TrkA, TrkC in pLKO and sh YKL-40 exosomes. EGFRvIII was decreased in sh YKL-40 exosomes, TrkA was undetected and TrkC was not changed.

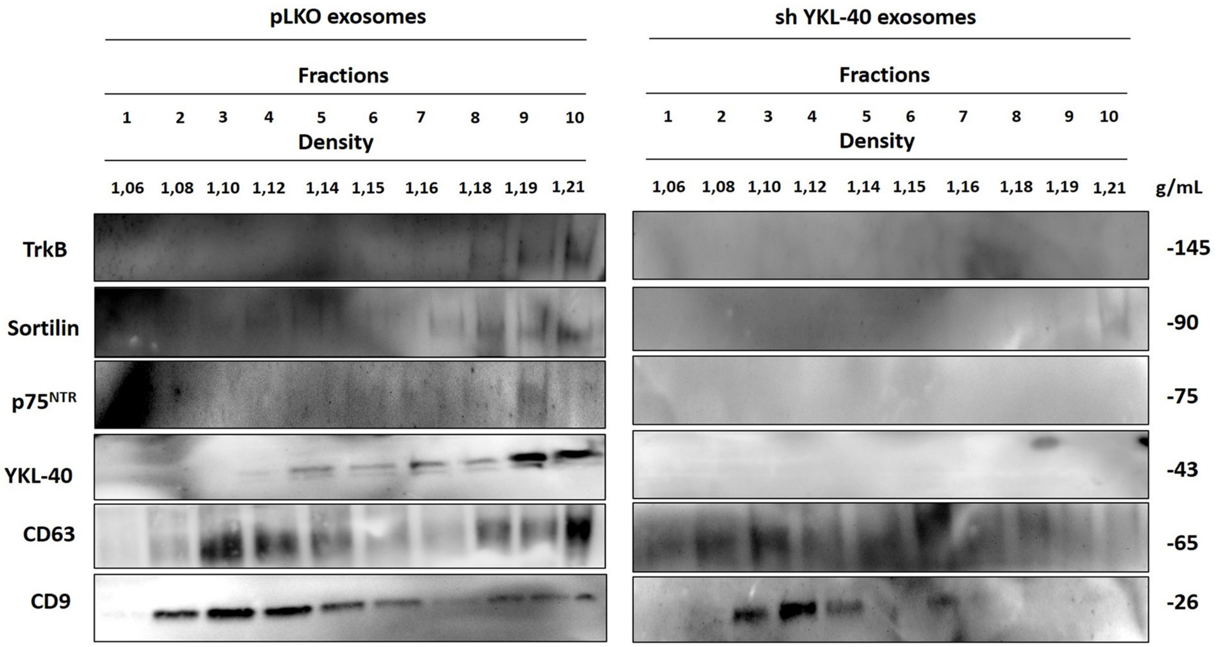

Supplementary Figure S4: YKL-40, TrkB, sortilin, p75^{NTR} display subtly different patterns after floatation on sucrose gradients. After isolation of exosomes by ultracentrifugation and sucrose cushion, pLKO exosomes and sh YKL-40 exosomes floated on a sucrose gradient (1.06 to 1.21 M). Density (g/mL) of each sucrose fractions measured by refractometry is indicated above the gel. Presence of characteristic exosome markers CD63, CD9 and expression of YKL-40, TrkB, sortilin, p75^{NTR} for each fraction of pLKO exosomes and sh YKL-40 exosomes were analyzed by western blotting.

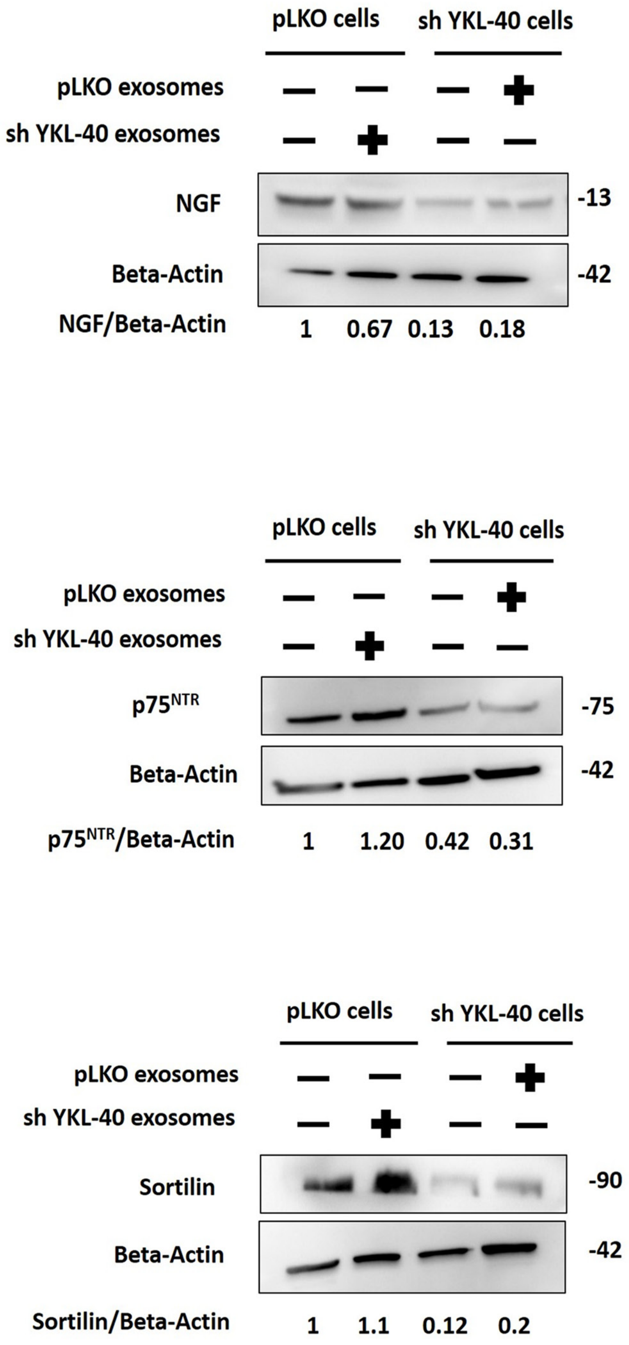

Supplementary Figure S5: NGF, P75^{NTR} and sortilin were not modified in sh YKL-40 cells by treatment with pLKO exosomes. Expression of NGF, p75^{NTR} and sortilin proteins in pLKO or sh YKL-40 cells after 24 hours co-culture with 30µg of pLKO or sh YKL-40 exosomes. Western blotting was normalized to β-actin.

Supplementary Table S1: Sequences of primers and probes used in qPCR

See Supplementary File 1
