# Supplementary material for: TrkB-containing exosomes promote the transfer of glioblastoma aggressiveness to YKL-40-inactivated glioblastoma cells
Source: Oncotarget. 2016 Jul 2;7(31):50349–64. doi: 10.18632/oncotarget.10387 (PMC5226587; doi:10.18632/oncotarget.10387)
Supplement: Supplementary file 2 [file oncotarget-07-50349-s002.docx]

**Supplementary Table S1 Sequences of primers and probes used in qPCR**

| **Amplified product** | **Accession number** | **Forward** | **Reverse** | **Length** | **probes** |
| --- | --- | --- | --- | --- | --- |
| **BDNF** | **NM_170735.5** | ***CAAAACGAAGGCCTCTGAAG*** | ***GGCTATGTGGAGTTGGCATT*** | **123** | ***ATTTCTGAGTGGCCATCCCAAGGTCTAG*** |
| **NGF** | **NM_002506.2** | ***AGCTTTCTATCCTGGCCACA*** | ***ATACAGGCGGAACCACACTC*** | **102** | ***AGGTGCATAGCGTAATGTCCATGTTGTTCT*** |
| **NTF3 variant 1** | **NM_001102654.1** | ***GTGGGGGAGACTTTGAATGA*** | ***GGTGAACAAGGTGATGTCCA*** | **127** | ***GCCATGGTTACTTTTGCCACGATCTTAC*** |
| **Sortilin** | **NM_002959.6** | ***AGCCAGTGGGTCTCCTACAC*** | ***CACTCCACAGACCCTGAAGA*** | **104** | ***TCCTTGAAAGGAACTGTGAAGAGAAGGACT*** |
| **TrkB FL (variant a NTRK2)** | **NM_006180.4** | ***CTGGTGAAAATCGGGGACT*** | ***AGGAAATTCACGACGGAAAG*** | **137** | ***TGTACAGCACTGACTACTACAGGGTCGGTG*** |
| **TrkB T1 (variant b NTRK2)** | **NM_001007097** | ***AAGATCCCACTGGATGGGTA*** | ***GGAAGTGCTGCTTATCTGGG*** | **138** | ***ATAAAGGAAAAGACAGAGAAAGGGGCTGTG*** |
| **TrkC (NTRK3)** | **NM_001012338.2** | ***TGAGAACCCCCAGTACTTCC*** | ***GCTCCCTCACCCAGTTCTC*** | **111** | ***CGTATGTGCAGCACATTAAGAGGAGAGACA*** |
| **YKL-40** | **NM_001276.2** | ***AAGCAAGGTGCAGTACCTGA*** | ***CTTGATGGCATTGGTGAGAG*** | **127** |  |
| **P75NTR (NGFR)** | **NM_002507.3** | ***CACCACCGACAACCTCATC*** | ***AAGCAGAACAAGCAAGGAGC*** | **117** | ***CCTACATAGCCTTCAAGAGGTGGAACAGCT*** |
| **GAPDH** | **NM_002046.5** | ***GGGTGGAATCATATTGGAACATG*** | ***CCAAATCCGTTGACTCCGAC*** | **142** |  |
